# Prosaposin knockdown in Caco-2 cells decreases cellular levels of coenzyme Q10 and ATP, and results in the loss of tight junction barriers

**DOI:** 10.3164/jcbn.16-32

**Published:** 2016-12-06

**Authors:** Misato Kashiba, Masayuki Terashima, Tomofumi Sagawa, Shinichi Yoshimura, Yorihiro Yamamoto

**Affiliations:** 1 School of Bioscience and Biotechnology, Tokyo University of Technology, 1404-1 Katakura-cho, Hachioji-shi, Tokyo 192-0982, Japan

**Keywords:** coenzyme Q10, prosaposin, Caco-2, transepithelial electrical resistance

## Abstract

Coenzyme Q10 (CoQ10) is a key component of the mitochondrial electron transfer chain and is one of the most important antioxidants. We previously found that glycoprotein prosaposin (Psap) binds CoQ10 in human cells. Although Psap is expressed in the intestines, its role in the gastrointestinal tract is not clear. To elucidate the role of Psap in the intestines, we established Psap knockdown (KD) Caco-2 cells, which are an intestinal epithelial cell line. Cellular CoQ10 levels decreased significantly in Psap KD Caco-2 cells as compared to parental Caco-2 cells. Cellular ATP levels also decreased significantly in Psap KD Caco-2 cells as compared to parental Caco-2 cells. Lower ATP levels in the intestines have been reported to result in the failure of tight junction formation. As expected, Psap KD Caco-2 monolayers did not produce transepithelial electrical resistance, while parental Caco-2 monolayers did. Moreover, a fluorescent dye, lucifer yellow, leaked out through Psap KD Caco-2 monolayers, whereas it did not through parental Caco-2 monolayers. These results indicate that Psap is essential to maintain cellular levels of CoQ10 and ATP, and consequently to form tight junctions in the gastrointestinal tract.

## Introduction

Prosaposin (Psap) is a multifunctional glycoprotein that exists as the lysosomal precursor of four small sphingolipid activator proteins, known as saposin A, B, C, and D.^(1)^ These four saposins are homologous to each other, having six conserved cysteines and one common glycosylation site. Psap also exists as a secreted protein, which has been found in various bodily fluids such as milk, serum, and seminal fluid.^(2)^ We previously reported that Psap and saposin B (SapB) bind and transfer coenzyme Q10 (CoQ10).^(3)^ We also reported that the loss of Psap in HepG2 cells decreases cellular CoQ10 levels, especially in mitochondria.^(4)^

CoQ10 is the main intrinsic lipid-soluble component of the energy-transducing membranes in mitochondria.^(5,6)^ Therefore, reduced levels of Psap may disrupt cellular energy metabolism via lowering CoQ10.

Although Psap is highly expressed in the gastrointestinal tract,^(7,8)^ its role there is not clear. It should be noted that lower than normal ATP levels in the intestines result in the failure of tight junction formation.^(9–11)^ Tsukamoto *et al.*^(9,10)^ reported that ATP depletion leads to a reversible decrease of transepithelial resistance (TER) in Madin-Darby canine kidney (MDCK) cell monolayers. They examined the effect of metabolic inhibitors (2-deoxy-d-glucose and antimycin A). Treatment of MDCK cells with these inhibitors led to a rapid decline in both ATP and TER to ~5% of initial values within 15 min. Dickman *et al.*^(11)^ reported that Rotavirus infection of Caco-2 cells reduced cellular ATP to 60% of controls at 24 h after infection, and caused disruption of tight junctions and loss of TER. Failure to form tight junctions in the gastrointestinal tract is relevant to various diseases such as bacterial gastritis, pseudomembranous colitis, Crohn’s disease, ulcerative colitis, celiac disease, and collagenous colitis.^(12)^

Therefore, we hypothesized that Psap might play a role in maintaining cellular ATP levels and tight junctions in the intestines. To elucidate the role of Psap in the intestines, we produced Psap knockdown (KD) Caco-2 cell lines and studied their tight junction formation.

## Material and Methods

### Cell culture

Cultures of Caco-2 cells (Riken, Tsukuba, Japan) were maintained in EMEM (Sigma, St. Louis, MO) supplemented with 10% fetal calf serum (HyClone, Thermo Fisher Scientific, Waltham, MA), 100 units/ml penicillin, 100 µg/ml streptomycin, and 100 µM non-essential amino acids (GIBCO, St. Louis, MO) at 37°C in a humidified atmosphere of 5% CO_2_ and 95% air.

### Design and synthesis of Psap miRNA, construction of vectors, and establishment of stable transfectants

 Pre-mi-RNA sequences for Psap were designed by Invitrogen’s RNAi Designer as reported previously.^(4)^ The sequences are as follows:

Psap miRNA1:

5'-TGCTGTACACTTCTCAGTTCCCAACAGTTTTGGCCACTGACTGACTGTTGGGATGAGAAGTGTA-3';

5'-CCTGTACACTTCTCATCCCAACAGTCAGTCAGTGGCCAAAACTGTTGGGAACTGAGAAGTGTAC-3';

Psap miRNA2:

5'-TGCTGTTGTCCTTCAGCATATCACCAGTTTTGGCCACTGACTGACTGGTGATACTGAAGGACAA-3';

5'-CCTGTTGTCCTTCAGTATCACCAGTCAGTCAGTGGCCAAAACTGGTGATATGCTGAAGGACAAC-3';

Psap miRNA3:

5'-TGCTGTCTCCAAGTAAACAAGGATCTGTTTTGGCCACTGACTGACAGATCCTTTTACTTGGAGA-3';

5'-CCTGTCTCCAAGTAAAAGGATCTGTCAGTCAGTGGCCAAAACAGATCCTTGTTTACTTGGAGAC-3';

Psap miRNA4:

5'-TGCTGATGACAGGGAGGTAGGAGTCCGTTTTGGCCACTGACTGACGGACTCCTCTCCCTGTCAT-3';

5'-CCTGATGACAGGGAGAGGAGTCCGTCAGTCAGTGGCCAAAACGGACTCCTACCTCCCTGTCATC-3'.

Synthesized complementary DNA oligos were annealed to generate double-stranded oligos and were cloned into linearized pcDNA^TM^6.2-GW/EmGFP-miR vector (Invitrogen, Carlsbad, CA) using T4 DNA ligase. All of the vectors were transformed into One Shot TOP10 Chemically Competent *E. coli* (Invitrogen), and colonies containing spectinomycin-resistant transformants were analyzed for the desired expression clones. The recombinant vectors were purified with a Qiagen Plasmid Midi Kit (Qiagen, Venlo, Netherlands).

Vectors with Psap miRNA1, Psap miRNA2, Psap miRNA3, and Psap miRNA4 were mixed and transfected into Caco-2 cells using Hylimax (Dojindo, Kumamoto, Japan) according to the manufacturer’s protocol. Two days later, cells were plated on 15-cm dishes. After blastcidin selection, clones were subjected to western blot analysis using an anti-SapB monoclonal antibody.^(3)^

### Western blotting analysis

Caco-2 cells were seeded in 24-well plates at a density of 30,000/cm^2^ and incubated for various times. At indicated times, cell media were removed and collected. Cells were washed two times with ice-cold PBS. Caco-2 cells in each dish were treated with lysis buffer [150 mM NaCl, 50 mM Tris-HCl, pH 7.4, 0.1% nonidet P-40 (Nakarai Tesuque, Tokyo, Japan), 0.1 mM EDTA, 1 mM phenylmethylsulfonyl fluoride, and 1 µg/ml of leupeptin, pepstatin A, *N*-tosyl-l-phenylalanyl chloromethyl ketone, and *N*-tosyl-l-lysyl chloromethyl ketone] for 1 h. Then the samples were collected and centrifuged at 15,000 × *g* for 10 min. Protein concentrations in the supernatants were measured with a Pierce BCA Protein Assay Kit (Thermo Fisher Scientific, Waltham, MA). Samples were separated by electrophoresis through a 15% SDS/polyacrylamide gel. After electrophoresis, proteins were transferred to PVDF membranes. The membranes were incubated with mouse anti-SapB IgG for 1 h at room temperature. Proteins were visualized with HRP-conjugated secondary antibodies (Bio-Rad Japan, Tokyo, Japan).

### Lipid analysis

Concentrations of CoQ10 and free cholesterol (FC) in cells were determined by HPLC, as reported previously.^(3)^ Briefly, cells were seeded in 24-well plates at a density of 30,000/cm^2^ and incubated for various times. The cell media were removed and the cells were washed with ice-cold PBS twice. Then, 400 µl of HPLC grade 2-propanol (Fisher Chemicals, Fairlawn, NJ) was added. Samples were collected and centrifuged. Extracts thus obtained were injected into the HPLC-ECD system. Mobile phase: 50 mM NaClO_4_ in methanol/2-propanol (7/3, v/v); flow rate: 1.0 ml/min; analytical column: KANTO RP-18 (L) GP, 5 µm × 150 mm × 4.6 mm (Kanto Chemical, Tokyo, Japan); post-reduction column: RC-10, 15 mm × 4 mm (IRICA, Kyoto, Japan). CoQ10 and FC concentrations were determined by an electrochemical detector (600 mV; NANOSPACE SI-1, Shiseido, Tokyo, Japan) and a UV detector (210 nm; SPD-10A, Shimadzu, Kyoto, Japan), respectively.

### Glucose consumption assay

Caco-2 cells were seeded in 24-well plates at a density of 30,000/cm^2^ and incubated for various times. At indicated times, cell media were collected. Glucose concentration in the medium was measured by a Glucose Assay Kit (Wako, Osaka, Japan).

### ATP analysis

Caco-2 cells were seeded at a density of 80,000 cells/cm^2^ and were grown as confluent monolayers on cell culture inserts (0.45 µm pore size, Millicell-HA, PIHA-01250; Merck Millipore, Billerica, MA). ATP was measured at day 21 after seeding. Cellular ATP concentrations were measured by CellTiter-Glo (Promega, Madison, WI).

### Transepithelial electrical resistance

Caco-2 cells were seeded at a density of 80,000 cells/cm^2^ and were grown as confluent monolayers on cell culture inserts (0.45 µm pore size, Millicell-HA, PIHA-01250). TER was measured at days 2, 4, 8, 12, 14, 18, and 29 with a Millicell-ERS (Millipore). Values were corrected for background resistance (130 Ω) and normalized to surface area (0.6 cm^2^).

### Permeability measurements

We determined the permeability of Caco-2 cell monolayers by measuring the transepithelial passage of a fluorescent dye, lucifer yellow (Invitrogen).^(13)^ Lucifer yellow is a fluorescein dye with a molecular weight of 444. Caco-2 cells (48,000 cells/well) were harvested on cell culture inserts. Cells were washed with Hank’s Balanced Salt Solution (HBSS, Invitrogen) twice. Then, HBSS (600 µl) was added to the basolateral side chamber and HBSS containing 100 µM lucifer yellow (450 µl) was applied to the apical side chamber. Then, the cultures were placed on a shaker in a 5% CO_2_ incubator at 37°C to ensure adequate mixing. Samples (100 µl) of the basolateral chambers were analyzed for fluorescence using a fluorescence microplate reader (Tecan, Zurich, Switzerland) at an excitation wavelength of 485 nm and an emission wavelength of 535 nm.

### Statistical analyses

Data were analyzed by Student’s *t* test, and values are means ± SD. *, **, and *** indicate significant differences (*p*<0.05, 0.01, and 0.001, respectively) between the two groups.

## Results and Discussion

### Silencing of Psap protein in Caco-2 cells

**Fig. 1:**
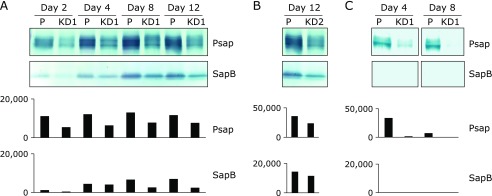
Detection of prosaposin (Psap) and saposin B (SapB) by using monoclonal anti-sapB IgG. (A) Levels of Psap and SapB in parental (P) and Psap knockdown (KD1) Caco-2 cells at days 2, 4, 8, and 12 after being seeded. (B) Levels of Psap and SapB at day 12 in parental (P) and Psap knockdown (KD2) Caco-2 cells. (C) Secretion of Psap and SapB into cell culture media at days 4 and 8. Data obtained from the densitometric analyses are also plotted.

Caco-2 cells were transfected with pcDNA^TM^ 6.2-GW/EmGFP-miR containing Psap miRNA. Blastcidine-resistant cell populations were subcloned: subcultures were derived from single cells. The EmGFP protein was visible under a fluorescence microscope. Western blotting was performed to evaluate the Psap knockdown. Psap and SapB protein levels increased with time in parental Caco-2 monolayers (Fig. 1A). Psap and SapB protein levels were lower in Psap KD cell line No. 1 (KD1) than those in parental cells at days 2, 4, 8, and 12 after seeding (Fig. 1A). Psap and SapB protein levels were also decreased in Psap KD cell line No. 2 (KD2, Fig. 1B). Psap exists in two forms, an intracellular form of 68 kDa and an extracellular form of 73 kDa.^(1)^ Psap is ubiquitously present in extracellular spaces such as blood and saliva.^(2)^ Caco-2 cells secrete Psap into their culture medium. Psap protein levels in conditioned cell culture media were lower for Psap knockdown cell line KD1 (Fig. 1C). SapB protein was not detectable in conditioned medium under the current experimental conditions. These results imply that Psap KD Caco-2 cell lines were successfully produced.

### Effect of Psap KD on cellular CoQ10 and ATP levels

**Fig. 2:**
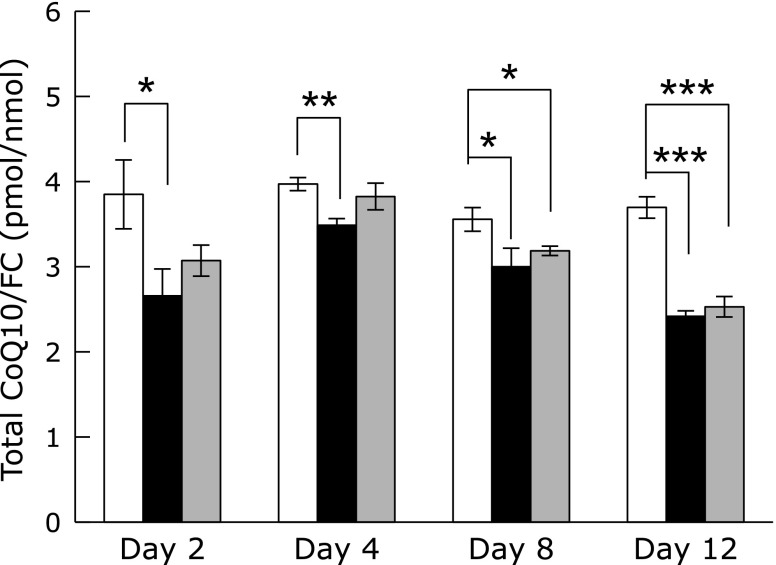
Molar ratios of coenzyme Q10 to free cholesterol (CoQ10/FC) in parental (white bar) and prosaposin knockdown (KD1: black bar, KD2: gray bar) Caco-2 cells. Data are expressed as mean ± SD. *p<0.05, **p<0.01, ***p<0.001 vs parental cell line.

 We previously reported that Psap and its derived protein SapB are CoQ10-binding proteins.^(3)^ Therefore, we measured cellular CoQ10 contents in parental and Psap KD Caco-2 cells. As shown in Fig. 2, ratios of CoQ10/FC in both KD1 and KD2 cells were lower than those in parental cells. The reason why CoQ10 divided by FC is to normalize CoQ10 content in cells since numbers of FC molecule are assumed to be constant in each Caco-2 cells. It should be noteworthy that overexpression of Psap in HepG2 cells resulted in an increase of CoQ10/FC ratio and Psap knockdown strains had decreased CoQ10/FC ratio as compared to that of parental HepG2 cells.^(4)^ Moreover, overexpression of Psap resulted in an increase of CoQ10 content not only in the mitochondria but also in plasma membranes, lysosomes, and nuclear membranes.^(4)^ These data clearly show that Psap regulates CoQ10 concentrations in cells probably by controlling the rate of CoQ10 transfer; i.e., high concentration of Psap accelerated CoQ10 transfer from its biosynthetic site to CoQ10-demanding membranes such as mitochondria, plasma, and nuclear membranes. This, in the end, promoted the CoQ10 synthesis. On the other hand, low concentration of Psap resulted in a decrease in the rate of CoQ10 transfer and its biosynthesis.

**Fig. 3:**
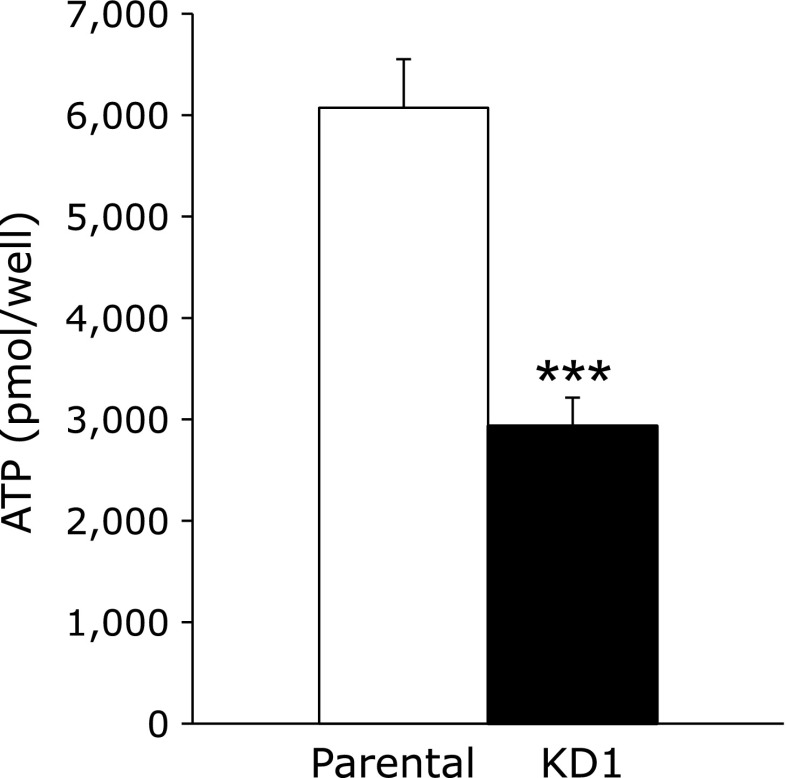
Cellular ATP contents in parental (white bar) and prosaposin knockdown (KD1, black bar) Caco-2 cells at day 21 after seeding. Data are expressed as mean ± SD. ***p<0.001 vs parental cell line.

Since CoQ10 is an important lipid molecule in mitochondrial electron transport to synthesize ATP,^(5)^ reduced levels of CoQ10 might reduce ATP in Psap KD Caco-2 cells. To validate this hypothesis, we next measured cellular ATP contents. As shown in Fig. 3, the ATP levels were significantly reduced in Psap KD Caco-2 cells as compared to parental cells. The ATP level was reduced to about 50% that of parental cells at day 21 after seeding.

### Cellular energy metabolism

**Fig. 4:**
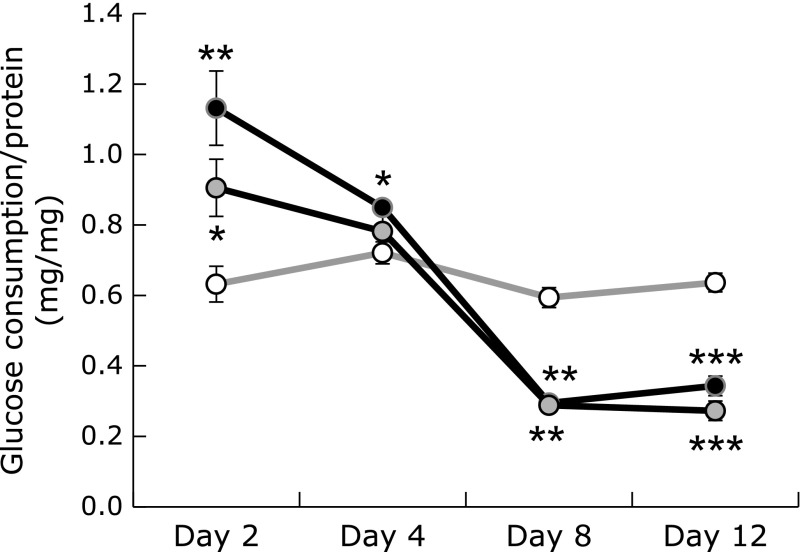
Glucose consumption by control (open circles) and prosaposin knockdown cell lines during 48 h. (KD1: black circles; KD2: gray circles). Data are expressed as mean ± SD. *p<0.05, **p<0.01, ***p<0.001 vs parental cell line.

We then measured the rate of cellular glucose consumption in Psap KD cell lines, in which CoQ10 concentrations and cellular ATP levels were reduced. As shown in Fig. 4, glucose consumption rates in KD1 and KD2 were accelerated at day 2. On the other hand, at days 8 and 12, glucose consumption rates decreased in Psap KD cells. The mechanism how glucose consumption rates were accelerated first and decreased later is unknown. But, above data show that glucose metabolism was disturbed in Psap KD cell lines, implying that cellular energy metabolism was perturbed.

### Effect of Psap KD on TER

**Fig. 5:**
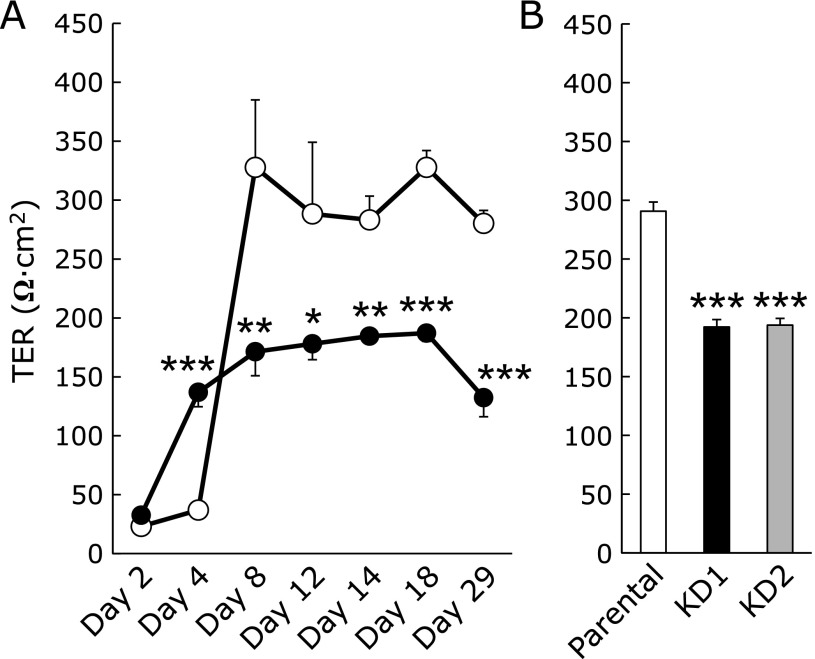
Effect of prosaposin knockdown on the barrier function of Caco-2 cells. (A) Time-course changes in transepithelial electrical resistance (TER) observed in parental (open circles) and KD1 (black circles) Caco-2 cells. (B) TER values at day 14 after seeding. Data are expressed as mean ± SD. *p<0.05, **p<0.01, ***p<0.001 vs parental cell line.

Lower than normal ATP levels have been reported to result in an increase in tight junction permeability of Caco-2 and MDCK cells.^(9–11)^ Tsukamoto and Nigam^(9,10)^ reported that chemical-induced ATP depletion reduces both ATP and TER levels to ~5% of the initial values in MDCK cells. Dickman *et al.*^(11)^ reported that Rotavirus infection reduces cellular ATP to 60% of control values and causes disruption of tight junctions in Caco-2 cells. Since the cellular ATP level in our Psap KD Caco-2 cells was reduced to 50% that of parental Caco-2 cells, we next examined the role of Psap on tight junction formation. We measured TER values in control and Psap KD Caco-2 cells. As shown in Fig. 5A, TER values of parental Caco-2 monolayers at days 2 and 4 were under 50 Ω · cm^2^. This value increased by day 8, averaged 328 Ω · cm^2^ after day 8, and remained constant by day 25, suggesting the formation of tight junctions in parental Caco-2 monolayers. On the other hand, KD1 Caco-2 monolayers failed to produce resistance. However, it is interesting that TER value of KD1 Caco-2 cells was greater than that of parental Caco-2 cells at day 4 (Fig. 5A). Similarly, glucose consumption rates in Psap KD Caco-2 cells were greater than those in parental Caco-2 cells at days 2 and 4 (Fig. 4). These two observations must be correlated but the underlying mechanism is not clear at this moment.

TER values of KD1 monolayers remained around 180 Ω · cm^2^. To confirm this result, the other Psap KD cell line, KD2, was seeded and TER was measured at day 14 (Fig. 5B). TER values of KD1 and KD2 monolayers were 192 Ω · cm^2^ and 194 Ω · cm^2^, indicating a failure to form tight junctions. The loss of TER suggests the disruption of barrier function for Psap KD cell lines.

### Permeability of Caco-2 monolayers

**Fig. 6:**
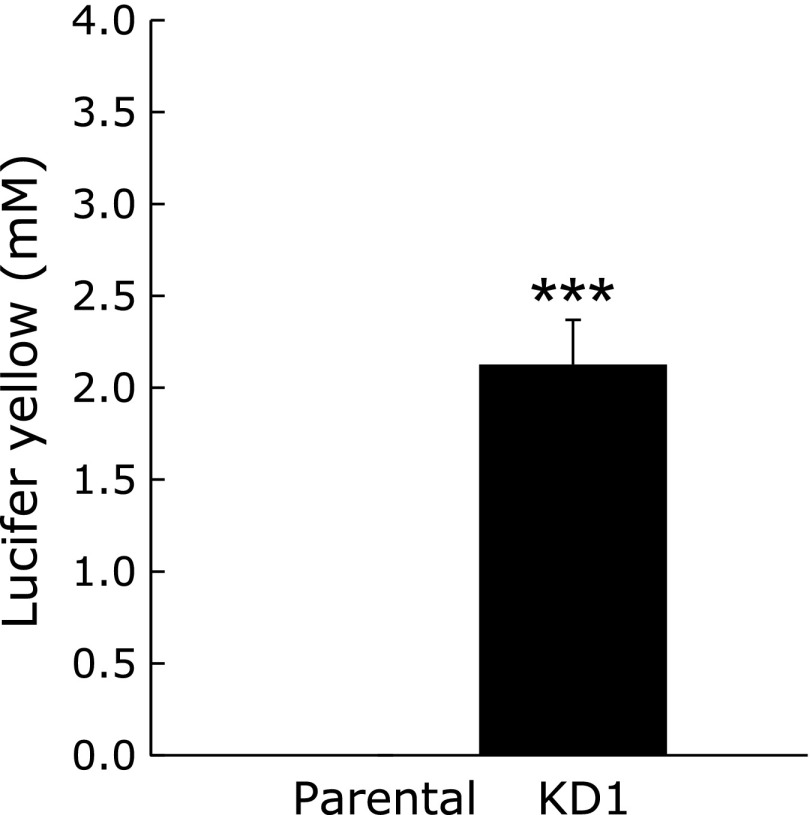
Effect of prosaposin knockdown on permeability of lucifer yellow through Caco-2 monolayers. Concentrations of lucifer yellow in basolateral media are expressed as mean ± SD. ***p<0.001 vs parental cell line.

We then assessed the permeability of Psap KD cell lines using the fluorescent dye lucifer yellow. When the barrier function is intact, lucifer yellow does not move from the apical to basolateral side. On the other hand, when barrier function is disrupted, lucifer yellow applied to the apical side moves to the basolateral side. Fig. 6 shows the concentration of lucifer yellow in the basolateral side of culture media. Lucifer yellow was applied to media on the apical side, and 2 h later, its concentration on the basolateral side was measured. As shown, no lucifer yellow was detected in basolateral side medium obtained from parental cells, suggesting the existence of a membrane barrier. On the other hand, significant amounts of lucifer yellow were detected in basolateral side medium obtained from Psap KD cell lines. This result shows that the barrier function was disrupted in Psap KD cells.

It is well known that ATP depletion causes the disruption of tight junction in Caco-2 and MDCK cells.^(9–11)^ Tsukamoto and Nigam observed that ATP repletion reforms tight junction in MDCK cells through tyrosine phosphorylation of tight junction proteins such as occludin.^(10)^ This should be the case in our Caco-2 cells and Psap can regulate tight junction barriers through the Psap-dependent regulation of CoQ10 and ATP concentrations.

## Conclusion

Knockdown of Psap in Caco-2 cell lines significantly decreased cellular CoQ10 and ATP levels as compared to parental cells, and resulted in disruption in tight junction formation. These results suggest that Psap plays an important role in maintaining cellular CoQ10 levels and in forming tight junctions in the gastrointestinal tract.

## Abbreviations

CoQ10: coenzyme Q10
KD: knockdown
MDCK: Madin-Darby canine kidney
Psap: prosaposin
SapB: saposin B
TER: transepithelial electrical resistance
